# Differences in health literacy and health-promoting behaviors by gender and sport type in collegiate athletes

**DOI:** 10.3389/fpubh.2026.1776062

**Published:** 2026-03-18

**Authors:** Hyung Ung Jung, Hee Ran Park, Jung-Min Lee, Hyun Chul Jung

**Affiliations:** 1 Department of Physical Education, Graduate School, Kyung Hee University, Yongin, Republic of Korea; 2 Graduate School of Education, Kyung Hee University, Yongin, Republic of Korea; 3 Department of Physical Education, College of Physical Education, Kyung Hee University, Yongin, Republic of Korea; 4 Department of Sports Coaching, College of Physical Education, Kyung Hee University, Yongin, Republic of Korea

**Keywords:** academic years, collegiate athletes, gender, health literacy, health-promoting behaviors, sports types

## Abstract

This study aims to investigate the influence of health literacy (HL) on health-promoting behaviors (HPB) in collegiate athletes. A total of 220 male and female students, including participants in individual sports, team sports, and non-athletes, were assessed. HL was measured using the European Health Literacy Survey Questionnaire, comprising three subdomains: perceptual, functional and linguistic HL. HPB was evaluated using a Health Promoting Lifestyle Profile-II. There were significant gender differences in overall HL (*P* = 0.038) and functional HL (*P* = 0.004). Team-sports athletes demonstrated significantly lower HL, particularly in perception and functional domains, compared with non-athletes (*P* < 0.001). HPB was positively correlated with HL (*P* < 0.001), perception HL (*P* < 0.001), and linguistic HL (*P* < 0.001). Regression analysis revealed that HL was a significant positive predictor of HPB (*P* < 0.001). These findings highlight that higher HL is associated with greater engagement in HPB among collegiate athletes. Tailored, sport-specific strategies that consider gender and type of sports may be essential to effectively enhance HL and promote health behaviors in this population.

## Introduction

Health literacy (HL) is increasingly recognized as a cornerstone of public health and an essential determinant of health outcomes and health-promoting behaviors (HPB). HL refers to an individual's ability to obtain, process, and apply health-related information in ways that enable informed decision-making and engagement in behaviors that contribute to long-term well-being (1). Conceptually, HL extends beyond basic reading and comprehension skills to include the motivation, cognitive ability, and confidence required to navigate health systems, evaluate health-related risks, and adopt preventive strategies. A growing body of literature demonstrates that higher HL is positively associated with healthier dietary practices, increased physical activity, reduced risky behaviors, and greater adherence to medical recommendations (2, 3). In contrast, individuals with low HL often struggle to understand health information, face difficulties in engaging with health services, and are less likely to participate in preventive health measures, which may contribute to health disparities and poorer quality of life. These findings underscore the importance of investigating HL across diverse populations, particularly in young adults transitioning into independence, such as collegiate athletes.

Collegiate athletes represent a unique subgroup for examining health literacy (HL). They experience the developmental challenges common to general collegiate students while simultaneously managing intensive training schedules, competitive stress, and academic demands (4). Within this environment, health-related decisions are frequently guided by coaches, which may limit athletes' opportunities to independently develop HL skills. In addition, collegiate athletes often emphasize performance-oriented health behaviors such as nutrition strategies and strength training while giving less consideration to health maintenance. Participation in collegiate sport has been further linked to risk-taking behaviors (5). These conditions underscore the need to study health literacy in college athletes, as it affects both competitive performance and long-term health after their athletic careers.

Within this context, it is important to consider the potential differences in HL across gender, type of sport participation, and academic year. Gender differences in HL have been reported in various populations, with women often demonstrating higher levels of HL than men (3). However, the extent to which this pattern holds among collegiate athletes remains less clear, particularly given that male athletes may have greater access to institutional resources in some sports type (6). Differences between team-sport and individual-sport athletes also warrant investigation. Team-sport athletes may experience greater reliance on group activities and coaching input, potentially limiting personal responsibility for health-related decision-making compared with individual-sport athletes who must take more self-organized action (7). Similarly, comparing athletes to their non-athlete peers provides insight into whether the structured lifestyle of sports participation enhances or constrains HL development. Academic year may further shape HL by reflecting accumulated exposure to university health resources or maturity in health decision-making, though previous research findings on this factor remain inconclusive.

Despite the theoretical relevance of HL, relatively few studies have systematically examined its relationship with HPB among collegiate athletes. Most existing research on HL has focused on general students, where HL has been linked to health behaviors such as balanced diet, stress management, and regular exercise (1, 8). However, the collegiate athletes experience distinct physiological, psychological, and social demands that may differentially affect both HL and HPB depending on group characteristics (9). For instance, athletes often exhibit higher levels of physical activity but may report lower attention to preventive health behaviors outside the athletic domain. Furthermore, HL is multidimensional, encompassing perceptual, functional, and linguistic domains (8). These subdomains may exert differential effects on health-promoting behaviors (HPB) among collegiate athletes. Previous studies, however, have primarily relied on unidimensional measures of health literacy or focused on isolated subgroup comparisons, such as gender or type of sport participation. Accordingly, evidence remains limited regarding gender- and sport type–specific differences in multidimensional health literacy and their associations with health-promoting behaviors.

Therefore, this study investigated whether HL differs by gender, academic year, and type of sport participation, and whether HL and its subdomains predict HPB. This study hypothesized that a) HL and HPB would differ by gender, academic year, and type of sport participation, and b) higher levels of HL would be positively associated with HPB among collegiate athletes. Identifying the role of HL in shaping HPB among student-athletes will provide valuable evidence for designing individualized and sport-specific interventions aimed at strengthening HL and promoting health across diverse athlete populations.

## Methods

### Participants

The participants in this study were male and female collegiate athletes registered with the Korea Sports Association, as well as students majoring in physical education at the university. A total of 221 collegiate athletes and non-athletes participated in the study. Collegiate athletes included individual sports (Swimming, Diving, Artistic Swimming, Sailing, Speed Skating, Figure Skating, Speed Skating, Snowboard, Skiing, Cycling, Golf, Equestrian, Artistic Gymnastics, Gymnastics, Wrestling, Fencing, Shooting, Archery, Modern Pentathlon, Taekwondo, Bowling) and team sports (Curling, Ice Hockey, Field Hockey, Football, Baseball, Basketball, Rugby, Volleyball, Handball) athletes. After excluding one student due to incomplete data, 220 questionnaires were used for data analysis. Prior to the study participation, all participants were informed of the study's purpose and procedures, and they voluntarily provided the written informed consent. The study protocol was approved by the Institutional Review Board of Kyung Hee University (IRB No.: KHGIRB-24-031). The general characteristics of the participants are presented in Table 1.

**Table 1:** General characteristics of participants (*N* = 220).

**Variables**	***M* ±*SD***	**% (*N)***
Age (yrs)	21.4 ± 2.23	
Height (m)	1.70 ± 0.10	
Weight (kg)	69.0 ± 15.17	
BMI (kg/m^2^)	23.0 ± 3.39	
**Gender**		
Male		59.5 (131)
Female		40.5 (89)
**Grade**		
Freshman		25.0 (55)
Sophomore		26.8 (59)
Junior		25.5 (56)
Senior		22.7 (50)
**Sports type**		
Individual Sports		32.3 (71)
Team Sports		34.5 (76)
Non–athlete student		33.2 (73)

M, Mean; SD, Standard deviation; BMI, Body mass index.

### Questionnaire

To achieve the purpose of this study, data were collected using a structured questionnaire consisting 96 items that included general characteristics, health literacy, and health-promoting behaviors (Table 2). Participants received a paper-based self-report questionnaire in a face-to-face setting under the supervision of a researcher who provided standardized instructions. The data were collected between March and June of 2024. To minimize the potential bias, anonymity was ensured, and participants were informed that their responses would not lead to any negative consequences.

**Table 2:** Measurement variables and instruments.

**Categories**	**Questionnaire**	**Number (96)**
General characteristics	Gender, Age, Grade, Sport type, Height, Weight, Level of health interest, Exercise time, Smoking status, Alcohol consumption frequency, Subjective health status, Health management method, Source of acquired health information, Type of acquired health information, Average sleep duration, Hospitalization status and current medical history	17
Health literacy	Perception domain: Subjective perception of understanding the health literacy	1
	Functional domain: Disease prevention (9 items), Health promotion (7 items)	16
	Linguistic domain: Access, understanding, appraisal, and application of health information	8
Health–promoting behaviors	Health responsibility (9 items), physical activity (8 items), Nutrition (9 items), mental growth (9 items), interpersonal relationships (9 items), stress management (8 items)	52

#### General characteristics

The general characteristics consisted of 17 items, including sex, age, academic year, sport, height, weight, interest in health, time spent exercising, smoking status, frequency of drinking, subjective health status, methods of health management, sources of health information obtained, types of health information obtained, average sleep duration, history of hospitalization, and current medical conditions.

#### Health literacy

The HL items consisted of three subdomains: perception of HL, functional HL, and linguistic HL. All items were rated on a 5-point Likert scale. 1 point for “Not at all,” 2 points for “No,” 3 points for “Neutral,” 4 points for “Yes,” and 5 points for “Very much so” (10).

- Perception domain: Perception HL was assessed using one item that asked about subjective awareness of health literacy. Each item was rated on a 5-point Likert scale: One point was assigned to the response “Not at all,” two points to “No,” three points to “Neutral,” four points to “Yes,” and five points to “Very much so” [7].
- Functional domain: Functional HL was measured using a tool revised and supplemented by (10), based on Jang's (2017) health literacy instrument (10, 11). Choi's revision removed items that were difficult to solve using mental calculation answer mentally. This questionnaire's reliability was reported as Cronbach's α = 0.760 (10). To account for potential statistical errors resulting from intuitive responses, a “Don't know” option was included for each item. The functional health literacy questionnaire consisted of 16 items, with respondents receiving 1 point for correct answers and 0 points otherwise (10).
- Linguistic domain: Linguistic HL was measured using a revised and supplemented tool by (10). This tool was based on a shortened version of the Korean adaptation of the European Health Literacy Survey Questionnaire (HLS-EU-Q47) and previous studies that condensed eHealth items (10, 12). Linguistic HL was divided into four subdomains, and the Cronbach's alpha coefficients for each were as follows. Accessing health information α = 0.825. Understanding health information α = 0.824. Appraising health information α = 0.822. Applying health information α = 0.794 (10).

Scores for each HL subdomain were calculated using the corresponding scoring procedures, with higher scores indicating higher levels of health literacy. Score ranges varied by subdomain: perceptual HL ranged from 1 to 5, functional HL from 0 to 16, and linguistic HL was calculated as a mean score ranging from 1 to 5. Perceptual HL was assessed using a single item on a 5-point Likert scale, functional HL was computed as the sum of correct responses, and linguistic HL represented the mean of item responses within each subdomain.

#### Health promoting behaviors

HPB were measured using a revised and supplemented tool by Hwang & Kang (2019), based on a questionnaire adapted by Seo (2001) from the Health Promoting Lifestyle Profile-II (HPLP-II), which was developed by Walker and Hill-Polerecky (1996) (13–15). The reliability of the revised questionnaire was reported as Cronbach's α = 0.93 (13). The questionnaire consisted of 52 items across six subdomains: health responsibility (nine items), physical activity (eight items), nutrition (nine items), spiritual growth (nine items), interpersonal relations (nine items), and stress management (eight items). Each item was rated on a 4-point Likert scale: One point for “Never,” two points for “Sometimes,” three points for “Often,” and four points for “Always” (13).

Scores for health-promoting behaviors (HPB) were calculated according to established scoring procedures, with higher scores indicating more frequent engagement in HPB. Total HPB scores ranged from 52 to 208, and subdomain scores were obtained by summing item responses within each respective domain.

### Statistical analyses

All statistical analyses were conducted using R Studio (RStudio, Boston, MA, USA). Descriptive statistics are presented as means and standard deviations (SD). Data normality was assessed using the Shapiro–Wilk test, and non-parametric statistical methods were applied accordingly. Group differences in health literacy (HL) and health-promoting behaviors (HPB) were examined using the Mann–Whitney U test. Differences according to academic year and type of sport participation were assessed using the Kruskal–Wallis test, followed by *post hoc* pairwise comparisons when significant effects were observed. Associations between HL and HPB were examined using Spearman's rank correlation coefficients. Simple linear regression analysis was performed to examine whether HL predicted HPB. One participant with incomplete data was excluded using complete-case analysis (listwise deletion). Statistical significance was set at α = 0.05.

## Results

Table 3 presents the comparisons of HL and HPB between male and female collegiate students. Female students demonstrated significantly higher overall HL than male students (*p* = 0.038). Among the HL subdomains, functional HL was also significantly higher among female students (*p* = 0.004). No significant gender differences were observed in perceptual HL or linguistic HL. Interestingly, HPB did not differ significantly between male and female students, despite differences in HL.

**Table 3:** Health literacy and health-promoting behaviors by gender.

**Variables**	**Male (*N* = 131)**	**Female (*N* = 89)**	***U-*value**	***p-*value**
Health literacy	40.1 ± 8.40	42.3 ± 7.63	6791.5	0.038
Perception health literacy	1.6 ± 0.80	1.9 ± 1.04	6399.5	0.178
Functional health literacy	11.5 ± 3.88	13.2 ± 2.57	7150.5	0.004
Linguistic health literacy	27.0 ± 6.30	27.3 ± 6.03	5924.0	0.838
Health–promoting behaviors	136.5 ± 23.95	134.2 ± 25.71	5517.0	0.500

Data are presented as mean ± standard deviation.

Table 4 presents the differences in HL and HPB across academic years. There were no significant differences in overall HL or HPB among freshmen, sophomores, juniors, and seniors.

**Table 4:** Health literacy and health-promoting behaviors by academic year.

**Variables**	**Freshman (*N* = 55)**	**Sophomore (*N* = 59)**	**Junior (*N* = 56)**	**Senior (*N* = 50)**	***χ^2^***	***p-*value**
Health literacy	40.6 ± 8.21	40.2 ± 7.15	41.9 ± 7.96	41.4 ± 9.46	3.218	0.359
Perception health literacy	1.7 ± 0.84	1.6 ± 0.81	1.9 ± 0.99	1.8 ± 1.00	1.754	0.625
Functional health literacy	12.2 ± 3.68	12.3 ± 3.08	12.3 ± 3.49	11.8 ± 3.86	0.534	0.911
Linguistic health literacy	26.7 ± 6.23	26.3 ± 5.60	27.8 ± 5.67	27.9 ± 7.24	3.412	0.332
Health–promoting behaviors	134.1 ± 25.26	133.5 ± 23.86	139.9 ± 21.54	134.9 ± 28.08	2.187	0.535

Data are presented as mean ± standard deviation.

Table 5 presents difference in HL and HPB by type of sport participation. Team-sport athletes demonstrated significantly lower HL compared with both individual-sport athletes and non-athlete students (*p* < 0.001). Within the HL subdomains, team-sport athletes scored lower on perceptual HL (*p* = 0.006) and functional HL (*p* < 0.001) relative to non-athletes, whereas no significant differences observed in linguistic HL. HPB did not differ significantly by type of sports participation.

**Table 5:** Health literacy and health-promoting behaviors by type of sport participation.

**Variables**	**Individual (*N* = 71)**	**Team (*N* = 76)**	**Non-athlete (*N* = 73)**	***χ^2^***	***p-*value**
Health literacy	42.6 ± 7.94^b^	38.6 ± 7.37^a^	42.2 ± 8.66^b^	15.245	<0.001
Perception health literacy	1.7 ± 0.93^ab^	1.5 ± 0.79^a^	2.0 ± 0.96^b^	10.326	0.006
Functional health literacy	12.2 ± 3.52^ab^	10.9 ± 3.82^a^	13.4 ± 2.64^b^	18.027	<0.001
Linguistic health literacy	28.5 ± 6.10	26.1 ± 6.03	26.9 ± 6.25	4.031	0.133
Health–promoting behaviors	139.5 ± 24.52	134.9 ± 25.56	132.5 ± 23.60	1.187	0.552

Data are presented as mean ± standard deviation.

Different letters indicate differences, identical letters indicate no difference.

**Figure 1:**
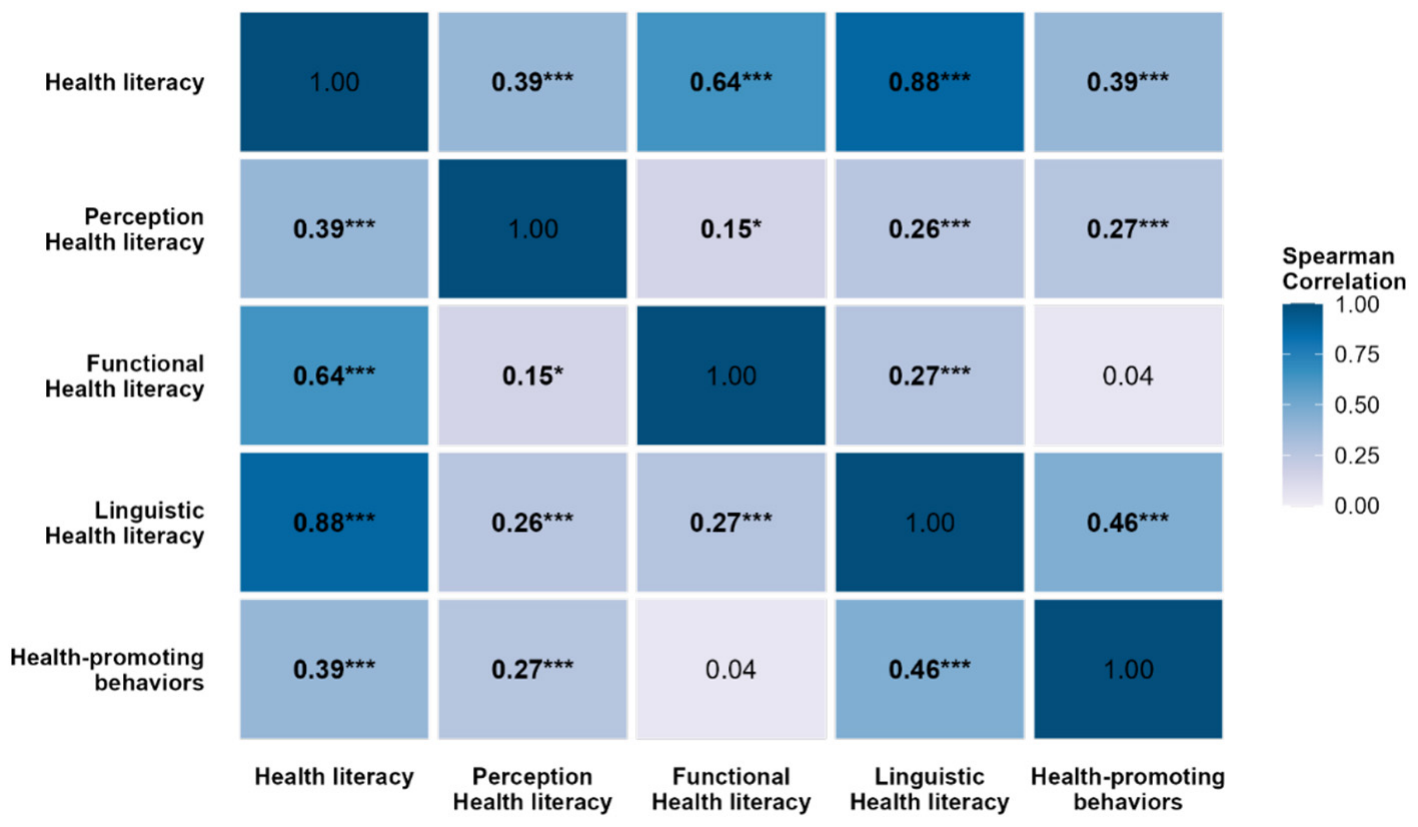
Correlation between health literacy and health-promoting behaviors. The color scale represents correlation coefficients; darker blue indicates stronger positive associations. ^*^p < 0.05, ^**^p < 0.001, ^***^p < 0.001.

The results of the correlation analysis are presented in Table 6 and Figure 1. Significant positive associations were observed between HPB and overall HL (*r* = 0.386, *P* < 0.001), perceptual HL (*r* = 0.270, *P* < 0.001), and linguistic HL (*r* = 0.462, *P* < 0.001). In contrast, functional HL was not significantly associated with HPB (*r* = 0.044, *p* = 0.520).

**Table 6:** Correlations between health literacy and health-promoting behaviors.

**Variables**	**Health literacy**	**Perception health literacy**	**Functional health literacy**	**Linguistic health literacy**
Health –promoting behaviors	0.386^***^	0.270^***^	0.044	0.462^***^

^*^p < 0.05, ^**^p < 0.01, ^***^p < 0.001.

**Figure 2:**
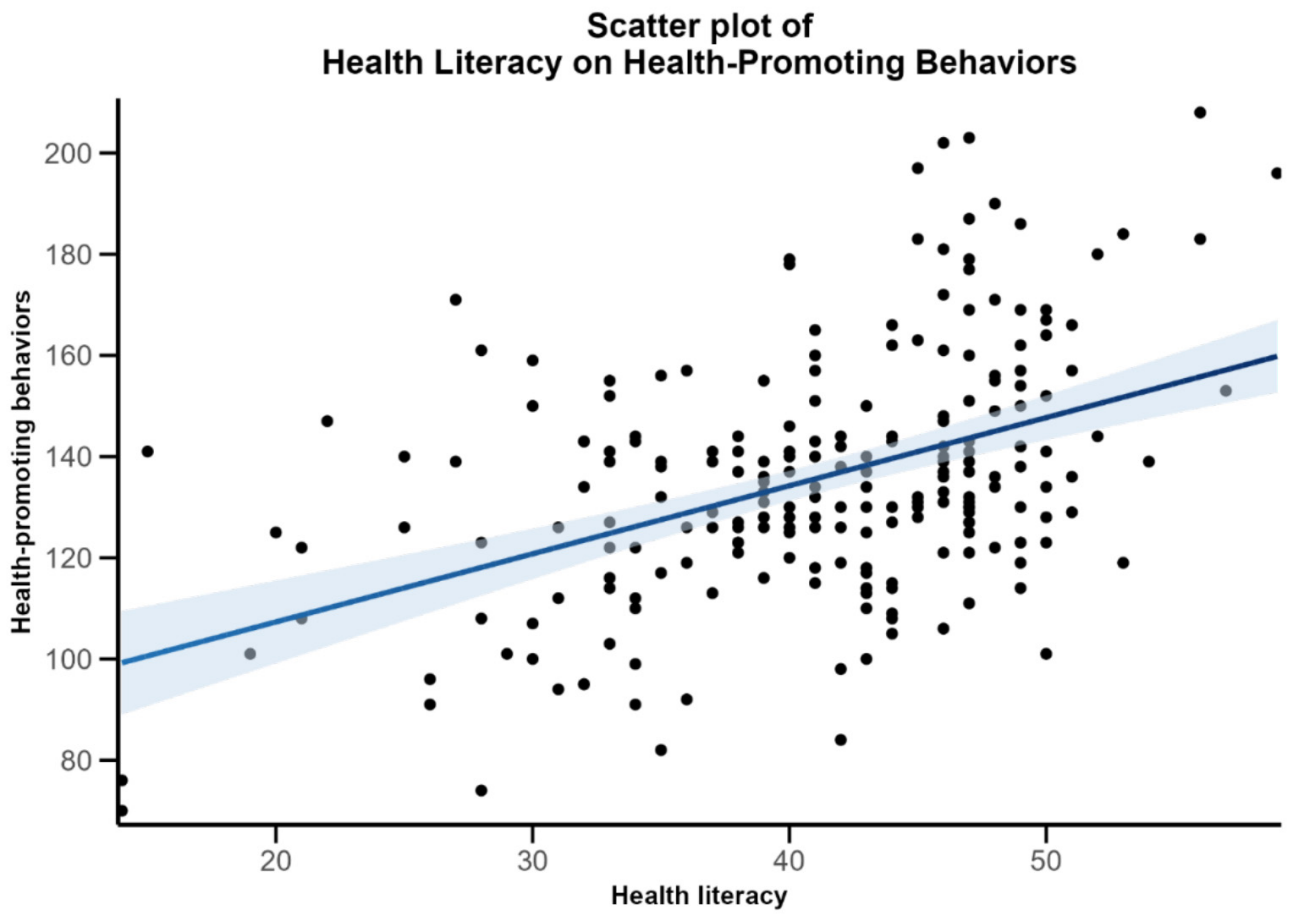
Scatter plot of health literacy and health-promoting behaviors. The shaded area represents the 95 % confidence interval.

The regression analysis revealed that HL was a significant predictor of HPB. The model accounted for approximately 20% of the variance in HPB, F (1, 218) = 53.92, *P* < 0.001, *R*^2^ = 0.20. The regression coefficient (β = 0.45) suggests that for every one-unit increase in HL, HPB increases by approximately 1.35 points, highlighting the practical significance of HL in shaping lifestyle practices. Figure 2 demonstrates the association between HL and HPB.

## Discussion

### Group differences in health literacy and health-promoting behaviors

This study examined the relationship between health literacy (HL) and health-promoting behaviors (HPB) among collegiate athletes, with comparisons by gender, academic year, and sport type. Female students demonstrated significantly higher overall HL and functional HL than male students, consistent with prior evidence that women engage more actively with health information and healthcare services (16, 17). According to Statistics Korea's 2024 data on healthcare service utilization rates, females participated at a higher rate than males, at 77.5% and 68.2%, respectively (17). There may also be differences in the purpose of internet use between male and female students. Male students reportedly use the internet primarily for entertainment and leisure, whereas female students use it more for maintaining interpersonal relationships and educational purposes (18). This trend may provide female students with more opportunities to search for and use health information, positively affecting health literacy (18). However, no difference in HL was observed by academic year. While previous studies demonstrated that HL tends to be higher among students in health-related majors (19) or those who have completed more semesters (20), our students appear to be limited to disciplines directly linked to public health and medicine. Collegiate athletes primarily focus on exercise and performance, which may have less opportunity to acquire health knowledge, thus HL may be more influenced by internal motivation and personality traits than academic alone. Yet, no gender differences in HPB were observed, suggesting that greater HL does not always translate into behavioral practice, an inconsistency also noted in earlier reviews (21).

Another notable finding was that team-sport athletes scored lower in HL—particularly perceptual and functional HL—than individual-sport athletes and non-athletes. Unlike team athletes, individual athletes are not embedded within a team and do not receive a uniform education or management from coaches. As individual sport athletes bear full responsibility for their competitive outcomes, they are required to independently manage essential elements of their performance, including health, weight, and training. This sense of personal accountability may foster more proactive and structured approaches to maintaining their health. In contrast, athletes in team sports train and compete within environments where team performance takes precedence over individual outcomes. Consequently, they may depend more on external regulation by coaches or team systems, rather than relying on self-directed management, which is more typical of individual sport athletes (22).

### Association between health literacy and health-promoting behaviors

Correlation and regression analyses further clarified these associations. HL, perceptual HL, and linguistic HL were positively correlated with HPB, while functional HL showed no significant relationship. These results suggest that higher-order HL competencies, such as critical appraisal of health information and effective communication, are more influential in driving behavioral engagement than basic comprehension skills. The regression analysis confirmed HL as a significant predictor of HPB, explaining 20% of the variance. These findings align with previous research indicating that higher HL levels positively influence health behavior by enabling individuals to seek, comprehend, and apply relevant health information (2). Individuals with higher HL can search for and use health information suited to them and maintain their health accordingly.

However, the non-significant contribution of functional HL raises questions about whether practical understanding alone is insufficient to promote behavior in the absence of awareness, motivation, and social support. A meta-study by Kim et al. (2023) (21) examining the relationship between HL and health behaviors reported cases in which groups with high HL rejected health behaviors, such as vaccination. This evidence that even individuals with high HL sometimes resist recommended health behaviors (21), reinforcing the need to consider both cognitive and contextual factors in HL research. Perceptual and linguistic HL may operate as higher-order competencies supporting the translation of health knowledge into behavioral practice. The significant positive correlations between perceived HL, linguistic HL, and HPB may stem from the learning and practice environments of physical education (PE) students. They often interact with health professionals, such as coaches, trainers, and medical professionals, and have repeated opportunities to listen to, ask questions about, and interpret health information. Additionally, learning HL concepts and HPB through physical education courses and practice may contribute to motivation for health behavior practices (23).

### Strengths and limitations

The strengths of this study include its focus on a rarely examined group, the use of validated instruments to assess both HL and HPB, and the comparison across gender, sport type, and non-athlete peers, which allowed nuanced insights into group-level differences. Nonetheless, several limitations must be noted. The cross-sectional design prevents causal inferences, and reliance on self-reported data introduces potential recall and social desirability bias. The sample was restricted to one university, limiting generalizability, and unmeasured factors such as socioeconomic status, academic major, and prior health education may have influenced the results. These strengths and weaknesses should be considered when interpreting the findings, but overall, the study provides new evidence on the multidimensional links between HL and HPB in collegiate athletes.

## Conclusions

The present study demonstrates that HL, particularly in the perceptual and linguistic domains, is positively associated with HPB among collegiate athletes. Female athletes demonstrated higher HL; however, this did not translate into corresponding differences in HPB, indicating that elevated HL alone may be insufficient for behavioral execution. No differences in HL were observed by academic year, suggesting that HL development in collegiate athletes is not driven solely by academic progression. Team-sport athletes exhibited lower levels of HL than individual-sport athletes, suggesting that reliance on externally regulated team systems may limit the development of self-directed HL competencies. Our findings highlight the need for gender-sensitive and sport-specific HL strategies that consider differences in responsibility and health information processing to promote sustainable health behaviors among collegiate athletes.

## Practical Implications

The findings have important implications for health promotion among collegiate athletes. Particularly, sport-specific strategies are needed, with a focus on strengthening HL in team-sport athletes, who appear more dependent on collective structures and less engaged in self-directed health management. Emphasis should be placed on developing perceptual and linguistic HL skills, which showed the strongest associations with HPB, through workshops, digital resources, and embedded health education in athletic training programs. For instance, mobile application-based nutrition education and workshop-based peer education programs enhance athletes' health-related knowledge, decision-making skills, and health behaviors (24, 25).

## Data Availability

The raw data supporting the conclusions of this article will be made available by the authors, without undue reservation.

## References

[1] SørensenK Van Den BrouckeS FullamJ DoyleG PelikanJ SlonskaZ . Health literacy and public health: a systematic review and integration of definitions and models. BMC Public Health. (2012) 12:1–13. doi: 10.1186/1471-2458-12-8022276600 PMC3292515

[2] Nutbeam D. Health literacy as a public health goal: a challenge for contemporary health education and communication strategies into the 21st century. Health Promot Int. (2000) 15:259–67. doi: 10.1093/heapro/15.3.259

[3] PaakkariL OkanO. COVID-19: health literacy is an underestimated problem. Lancet Public Health. (2020) 5:e249–50. doi: 10.1016/S2468-2667(20)30086-432302535 PMC7156243

[4] Divin AL. Mental Health in College Athletics: It's Time for Social Work to Get in the Game on JSTOR. Stillwater, OK: Oklahoma State University. (2009). 163.

[5] McLeodG O'ConnorS MorganD KountourisA FinchCF Fortington LV. Medical-attention injuries in community cricket: a systematic review. BMJ Open Sport Exerc Med (2020) 6:e000670. doi: 10.1136/bmjsem-2019-00067032231790 PMC7101051

[6] O'Connor JJ. The means to an end: an examination of gender inequality in athletic aid distribution and graduation rates. Sport Soc. (2021) 24:534–50. doi: 10.1080/17430437.2019.1679770

[7] Jonker L Elferink-Gemser MT Visscher C. Differences in self-regulatory skills among talented athletes: The significance of competitive level and type of sport. J Sports Sci. (2010) 28:901–8. doi: 10.1080/02640411003797157 20544490

[8] HLS-EU Consortium. Comparative report of health literacy in eight EU member states: the European Health Literacy Survey HLS-EU. (2012).

[9] Gomez J Bradley J Conway P. The challenges of a high-performance student athlete. Irish Educational Studies. (2018) 37:329–49. doi: 10.1080/03323315.2018.1484299

[10] Choi S-EA. Comparative study on health literacy between dietitians (Nutrition Teachers) and general adults. [Master's thesis]. Seoul: The Graduate School of Education, Chung-Ang University. (2023).

[11] Jang B. Development of korean adolescent health literacy scale (KHLS-Teen). Busan: The Graduate School, Pusan National University. (2017).

[12] FinbråtenHS Wilde-LarssonB NordströmG PettersenKS TrollvikA GuttersrudØ. Establishing the HLS-Q12 short version of the European Health Literacy Survey Questionnaire: Latent trait analyses applying Rasch modelling and confirmatory factor analysis. BMC Health Serv Res. (2018) 18:1–17. doi: 10.1186/s12913-018-3275-729954382 PMC6022487

[13] Hwang A-R Kang H-W. Influence of eHealth literacy on health promoting behaviors among university students. J Korean Soc Sch Health. (2019) 32:165–74. doi: 10.15434/kssh.2019.32.3.165

[14] Seo H. Construction of a health promoting behaviors model in the elderly. Seoul: Graduate School of Seoul National University. (2001).

[15] Walker SN Sechrist KR Pender NJ. Health Promotion Model - Instruments to Measure Health Promoting Lifestyle: Health-Promoting Lifestyle Profile [HPLP II] (Adult Version). (1995)

[16] Bertakis KD Azari R Helms LJ Callahan EJ Robbins JA. Gender differences in the utilization of health care services. J Fam Pract. (2000) 49:147–52. 10718692

[17] Statistics Korea. Social Survey—Health Part Raw Data Analysis. Daejeon: Statistics Korea (2024)

[18] BonaccorsiG GallinoroV GuidaA MorittuC AllodolaVF LastrucciV . Digital health literacy and information-seeking in the era of covid-19: gender differences emerged from a florentine university experience. Int J Environ Res Public Health. (2023) 20:2611. doi: 10.3390/ijerph2003261136767976 PMC9915269

[19] KühnL BachertP HildebrandC KunkelJ ReitermayerJ WäscheH . Health literacy among university students: a systematic review of cross-sectional studies. Front Public Health. (2022) 9:680999. doi: 10.3389/fpubh.2021.68099935127605 PMC8814326

[20] YangSC LuoYF ChiangCH. The associations among individual factors, ehealth literacy, and health-promoting lifestyles among college students. J Med Internet Res. (2017) 19:e5964. doi: 10.2196/jmir.596428073739 PMC5263862

[21] KimK ShinS KimS LeeE. The relation between ehealth literacy and health-related behaviors: systematic review and meta-analysis. J Med Internet Res. (2023) 25:e40778. doi: 10.2196/4077836716080 PMC9926349

[22] ŠagátP BartikP LazićA TohăneanDI KoronasV TurcuI . Self-Esteem, individual versus team sports. Int J Environ Res Public Health. (2021) 18:12915. doi: 10.3390/ijerph18241291534948525 PMC8701405

[23] Rubinelli S Schulz PJ Nakamoto K. Health literacy beyond knowledge and behaviour: letting the patient be a patient. Int J Public Health. (2009) 54:307–11. doi: 10.1007/s00038-009-0052-8 19641846

[24] AlKasasbeh WJ Amawi AT Al-Nawaiseh SJ Alshorman D Alshdaifat K Alawamleh T . Educational intervention using a mobile app to enhance sports nutrition knowledge and dietary habits in student-athletes: a randomized controlled trial. Front Educ. (2025) 10:1622166. doi: 10.3389/feduc.2025.1622166

[25] KneavelME ErnstW McCarthyKS. Randomized controlled trial of a novel peer concussion-education program for collegiate athletes. J Athl Train. (2020) 55:456–68. doi: 10.4085/1062-6050-0182.1932298143 PMC7249285

